# News from the forest of Mondulkiri: additions to the mosquito fauna of Cambodia (Diptera: Culicidae)

**DOI:** 10.1051/parasite/2025064

**Published:** 2025-12-02

**Authors:** Pierre-Olivier Maquart, Kimhuor Suor, Amélie Vantaux, Bros Doeurk, Kalyan Chhoy, Kimly Heng, Saoya Sen, Rina Chea, Kimsear Nov, Theary Keum, Sokkeang Leng, Sébastien Boyer

**Affiliations:** 1 Medical and Veterinary Entomology Unit, Institut Pasteur du Cambodge, Institut Pasteur International Network PO Box 983 Phnom Penh Cambodia; 2 Institut de Recherche pour le Développement, UMR-247 EGCE IRD, CNRS, Université Paris Saclay 91190 Gif-sur-Yvette Cedex France; 3 Forestry Administration, Ministry of Agriculture Forestry and Fisheries Cambodia; 4 Malaria Research Unit, Institut Pasteur du Cambodge PO Box 983 Phnom Penh Cambodia; 5 Emergence and Ecology of Arthropod-borne diseases, Institut Pasteur 25–28 Rue du Dr Roux 75015 Paris France

**Keywords:** Public health, Culicidae, Diversity, Arbovirus

## Abstract

Following the ongoing inventory efforts conducted in Cambodia by the Medical and Veterinary Unit of the Institut Pasteur du Cambodge, and after intensive collection work in the eastern part of the country, we report 20 new mosquito species from Cambodia. Among these new records, none are considered to be of medical importance. Along with these new records, and incorporating 2 recently described species, we provide an updated checklist of the Culicidae fauna of Cambodia, which now includes 312 species, 43 of which are known to be of medical importance and are involved in the transmission of pathogens.

## Abstract (fr)

Suite aux efforts d’inventaire menés au Cambodge par l’Unité Médicale et Vétérinaire de l’Institut Pasteur du Cambodge, et après un travail intensif de collecte dans la partie orientale du pays, nous rapportons 20 espèces de moustiques nouvelles pour le Cambodge. Parmi ces nouveaux signalements, aucun n’est considéré comme ayant une importance médicale. Avec ces nouveaux signalements, et en incorporant 2 espèces récemment décrites, nous fournissons une liste actualisée de la faune des Culicidae du Cambodge, qui comprend maintenant 312 espèces, dont 43 sont connues pour être d’importance médicale et impliquées dans la transmission d’agents pathogènes.

## Introduction

Mosquitoes play a crucial role in the circulation of infectious diseases. Climate and land-use changes, globalization of trade, and transportation significantly influence their spread and the pathogens they sometimes carry, contributing to the expansion of emerging or re-emerging diseases worldwide [8]. Understanding mosquito diversity and biology is essential to understand their role in the vectorial cycle and the potential risks they pose to human and animal health. Therefore, knowledge of the presence and current range of mosquito species in a given area is crucial to assess potential risks to health.

Between 2016 and 2020, over 230,000 specimens were collected as part of the collecting and taxonomic efforts undertaken by the medical and veterinary entomology unit of the Institut Pasteur du Cambodge. These collections made it possible to establish a preliminary checklist consisting of 20 genera and 290 species, with 49 species newly reported in Cambodia [8]. Since then, two new species have been described [3, 9]. Furthermore, following large-scale sampling conducted in the forests of Mondulkiri province in eastern Cambodia, where monthly collections took place for one year [4, 7], we were able to identify an additional 61,389 mosquitoes, from 16 genera and 123 species, enabling completion of the mosquito checklist of Cambodia previously published [8]. Mondulkiri is one of the largest provinces in Cambodia and hosts one of the last remaining pockets of malaria [11]. It is known for its rich natural resources, including wildlife and forests [2, 12]. However, deforestation is progressing at an alarming rate [1, 11, 23], due to large land concessions where companies acquire and convert vast forest areas for agriculture [1], “slash and burn” activities conducted by local communities to expand their agricultural parcels, and illegal logging [23]. This increase in forest activity and rapid environmental changes may alter the ongoing vectorial cycle between some mosquito species and their natural hosts. In this context, improving our knowledge of mosquito diversity and ecology is crucial to monitor changes in their bionomics and the impact these changes may have on the transmission of vector-borne diseases.

## Material and methods

### Study areas and specimen collection

Our study was conducted in the forested area of Roya commune, located in Kaoh Nheaek district, Mondulkiri province. The study was carried out at 16 different sites, including 3 in villages and 13 in forest areas over one year in 2020. More detailed information is available in [7]. Sampling occurred monthly for three consecutive days at each site. At each site, we used a human-baited double net trap to collect mosquitoes. This double net system consisted of an inner net (measuring 180 × 180 × 250 cm) completely closed to protect the human volunteer from mosquito bites, while the outer layer (200 × 200 × 250 cm) was suspended 20 cm above the ground to allow mosquitoes to fly inside the trap. For each site, two volunteers took turns in collecting mosquitoes and staying in the double-net system. Attracted adult mosquitoes were collected every hour for 15 min over 72 h (3 consecutive days).

### Morphological identification and nomenclature

Specimens were identified using diagnostic morphological characters [13–20], which were later confirmed by the examination of the original species descriptions. Identification were only performed on adult specimens. Only morphological identification was carried out. Voucher specimens were deposited in the collection of the Institut Pasteur du Cambodge, Phnom Penh, Cambodia.

The name abbreviations follow the taxonomic nomenclature of Knight & Stone [5, 6] and Reinert [21, 22]. For the names of the Aedini tribe, we followed the classification provided by Wilkerson *et al.* [24].

## Results

A total of 61,389 specimens were collected and 51,549 were identified to the species level. In total, 122 species from 16 genera were collected by the Malaria Research Unit of the Institut Pasteur du Cambodge in Mondulkiri province between 2020 and 2021 and identified by the medical and veterinary entomology unit. While 21 species were newly reported for the country, none of these species are of medical importance. The updated checklist of the mosquito fauna of Cambodia, now reaching 312 species, is presented in Table 1. All new records belong to Subfamily Culicinae Meigen, 1818, Tribe Aedini Neveu-Lemaire, 1902.

**Table 1:** Updated list of the Culicidae fauna of Cambodia.

**Subfamily Anophelinae Grassi, 1900**
**Genus *Anopheles* Meigen, 1818 (53 spp.)**
**Subgenus *Anopheles* Meigen, 1818 (28 spp.)**
*Anopheles* (*Anopheles*) *annandalei* Prashad, 1918 *Anopheles* (*Anopheles*) *argyropus* (Swellengrebel, 1914) *Anopheles* (*Anopheles*) *baezai* Gater, 1933 *Anopheles* (*Anopheles*) *barbirostris* van der Wulp, 1884 *Anopheles* (*Anopheles*) *barbumbrosus* Strickland & Chowdhury, 1927 *Anopheles* (*Anopheles*) *bengalensis* Puri, 1930 *Anopheles* (*Anopheles*) *campestris* Reid, 1962 (dubious) *Anopheles* (*Anopheles*) *crawfordi* Reid, 1953 *Anopheles* (*Anopheles*) *dissidens* Taai & Harbach, 2015 *Anopheles* (*Anopheles*) *donaldi* Reid, 1962 *Anopheles* (*Anopheles*) *hodgkini* Reid, 1962 *Anopheles* (*Anopheles*) *hyrcanus* (Pallas, 1771) (dubious) *Anopheles* (*Anopheles*) *insulaeflorum* (Swellengrebel & Swellengrebel de Graaf, 1920) *Anopheles* (*Anopheles*) *interruptus* Puri, 1929 *Anopheles* (*Anopheles*) *lesteri* Baisas & Hu, 1936 *Anopheles* (*Anopheles*) *letifer* Sandosham, 1944 *Anopheles* (*Anopheles*) *nigerrimus* Giles, 1900 *Anopheles* (*Anopheles*) *nitidus* Harrison, Scanlon & Reid, 1973 *Anopheles* (*Anopheles*) *peditaeniatus* (Leicester, 1908) *Anopheles* (*Anopheles*) *pursati* Laveran, 1902 *Anopheles* (*Anopheles*) *roperi* Reid, 1950 *Anopheles* (*Anopheles*) *saeungae* Taai & Harbach, 2015 *Anopheles* (*Anopheles*) *separatus* (Leicester, 1908) *Anopheles* (*Anopheles*) *sinensis* Wiedemann, 1828 *Anopheles* (*Anopheles*) *sintonoides* Ho, 1938 *Anopheles* (*Anopheles*) *umbrosus* (Theobald, 1903) *Anopheles* (*Anopheles*) *wejchoochotei* Taai & Harbach, 2015 *Anopheles* (*Anopheles*) *whartoni* Reid, 1963
**Subgenus *Cellia* Theobald, 1902 (25 spp.)**
*Anopheles* (*Cellia*) *aconitus* Dönitz, 1902 *Anopheles* (*Cellia*) *annularis* van der Wulp, 1884 *Anopheles* (*Cellia*) *baimaii* Sallum, Peyton & Wilkerson, 2005 *Anopheles* (*Cellia*) *culicifacies* Giles, 1901 *Anopheles* (*Cellia*) *dirus* Peyton & Harrison, 1979 *Anopheles* (*Cellia*) *epiroticus* Linton & Harbach, 2005 *Anopheles* (*Cellia*) *indefinitus* Ludlow, 1904 *Anopheles* (*Cellia*) *jamesii* Theobald, 1901 *Anopheles* (*Cellia*) *jeyporiensis* James, 1902 *Anopheles* (*Cellia*) *karwari* (James, 1903) *Anopheles* (*Cellia*) *kochi* Dönitz, 1901 *Anopheles* (*Cellia*) *maculatus* Theobald, 1901 *Anopheles* (*Cellia*) *minimus* Theobald, 1901 *Anopheles* (*Cellia*) *nivipes* (Theobald, 1903) *Anopheles* (*Cellia*) *notanandai* Rattanarithikul & Green, 1987 *Anopheles* (*Cellia*) *pallidus* Theobald, 1901 (dubious) *Anopheles* (*Cellia*) *pampanai* Büttiker & Beales, 1959 *Anopheles* (*Cellia*) *philippinensis* Ludlow, 1902 *Anopheles* (*Cellia*) *pseudojamesi* Strickland & Chowdhury, 1927 *Anopheles* (*Cellia*) *rampae* Harbach & Somboon, 2011 *Anopheles* (*Cellia*) *sawadwongporni* Rattanarithikul & Green, 1987 *Anopheles* (*Cellia*) *splendidus* Koidzumi, 1920 *Anopheles* (*Cellia*) *subpictus* Grassi, 1899 *Anopheles* (*Cellia*) *tessellatus* Theobald, 1901 *Anopheles* (*Cellia*) *vagus* Dönitz, 1902
**Subfamily Culicinae Meigen, 1818**
**Tribe: *Aedeomyiini* Theobald, 1901**
**Genus *Aedeomyia* Theobald, 1901**
**Subgenus *Aedeomyia* Theobald, 1901 (1 sp.)**
*Aedeomyia* (*Aedeomyia*) *catasticta* Knab, 1909
**Tribe: *Aedini* Neveu-Lemaire, 1902**
**Genus *Aedes* Meigen, 1818 (69 spp.)**
**Subgenus *Aedimorphus* Theobald, 1903 (9 spp.)**
*Aedes* (*Aedimorphus*) *alboscutellatus* (Theobald, 1905) *Aedes* (*Aedimorphus*) *caecus* (Theobald, 1901) *Aedes* (*Aedimorphus*) *culicinus* (Edwards, 1922) *Aedes* (*Aedimorphus*) *mediolineatus* (Theobald, 1901) *Aedes (Aedimorphus) nigrostriatus* (Barraud, 1927) **NEW RECORD** *Aedes* (*Aedimorphus*) *pampangensis* (Ludlow, 1905) *Aedes* (*Aedimorphus*) *pipersalatus* (Giles, 1902) *Aedes* (*Aedimorphus*) *taeniorhynchoides* (Christophers, 1911) *Aedes* (*Aedimorphus*) *vexans* (Meigen, 1830)
**Subgenus *Bothaella* Reinert, 1973 (3 spp.)**
*Aedes* (*Bothaella*) *eldridgei* Reinert, 1973 *Aedes* (*Bothaella*) *helenae* Reinert, 1973 *Aedes* (*Bothaella*) *kleini* (Reinert, 1973)
**Subgenus *Bruceharrisonius* Reinert, 2003 (2 sp.)**
*Aedes* (*Bruceharrisonius*) *aureostriatus* (Doleschall, 1857) *Aedes* (*Bruceharrisonius*) *greenii* (Theobald, 1903) **NEW RECORD**
**Subgenus *Christophersiomyia* Barraud, 1923 (3 spp.)**
*Aedes* (*Christophersiomyia*) *annulirostris* (Theobald, 1905) *Aedes* (*Christophersiomyia*) *ibis* Barraud, 1931 *Aedes* (*Christophersiomyia*) *thomsoni* (Theobald, 1905)
**Subgenus *Collessius* Reinert, Harbach & Kitching, 2006 (3 spp.)**
*Aedes* (*Collessius*) *elsiae* (Barraud, 1923) *Aedes* (*Collessius*) *macfarlanei* (Edwards, 1914) *Aedes* (*Collessius*) *pseudotaeniatus* (Giles, 1901)
**Subgenus *Danielsia* (Leicester, 1904) (1 sp.)**
*Aedes* (*Danielsia*) *albotaeniatus* (Leicester, 1904)
**Subgenus *Downsiomyia* Vargas, 1950 (8 spp.)**
*Aedes* (*Downsiomyia*) *albolateralis* (Theobald, 1908) *Aedes* (*Downsiomyia*) *ganapathi* Colless, 1958 **NEW RECORD** *Aedes* (*Downsiomyia*) *harinasutai* Knight, 1978 **NEW RECORD** *Aedes* (Downsiomyia) litoreus Colless, 1958 **NEW RECORD** *Aedes* (*Downsiomyia*) *mikrokopion* Knight & Harrison, 1988 *Aedes* (*Downsiomyia*) *nipponicus* La Casse & Yamaguti, 1948 **NEW RECORD** *Aedes* (*Downsiomyia*) *niveoides* (Barraud, 1934) *Aedes* (*Downsiomyia*) *novoniveus* Barraud, 1934 **NEW RECORD**
**Subgenus *Edwardsaedes* Belkin, 1962 (1 sp.)**
*Aedes* (*Edwardsaedes*) *imprimens* (Walkar, 1860)
**Subgenus *Fredwardsius* Reinert, 2000 (1 sp.)**
*Aedes* (*Fredwardsius*) *vittatus* (Bigot, 1861)
**Subgenus *Himalaius* Reinert, Harbach & Kitching, 2006 (1 sp.)**
*Aedes* (*Himalaius*) *gilli* (Barraud, 1924)
**Subgenus *Hulecoeteomyia* Theobald, 1904 (3 spp.)**
*Aedes* (*Hulecoeteomyia*) *chrysolineatus* (Theobald, 1907) *Aedes* (*Hulecoeteomyia*) *harveyi* (Barraud, 1923) *Aedes* (*Hulecoeteomyia*) *saxicola* Edwards, 1922
**Subgenus *Kenknightia* Reinert, 1990 (2 sp.)**
*Aedes* (*Kenknightia*) *dissimilis* (Leicester, 1908) *Aedes* (*Kenknightia*) *harbachi* Reinert, 1990 **NEW RECORD**
**Subgenus *Lorrainea* Belkin, 1962 (2 spp.)**
*Aedes* (*Lorrainea*) *amesii* (Ludlow, 1903) *Aedes* (*Lorrainea*) *fumidus* Edwards, 1928
**Subgenus *Mucidus* Theobald, 1901 (2 spp.)**
*Aedes* (*Mucidus*) *laniger* (Wiedemann, 1820) *Aedes* (*Mucidus*) *quasiferinus* Mattingly, 1961
**Subgenus *Neomelaniconion* Newstead, 1907 (1 sp.)**
*Aedes* (*Neomelaniconion*) *lineatopennis* (Ludlow, 1905)
**Subgenus *Ochlerotatus* (Skuse, 1889) (1 sp.)**
*Aedes* (*Ochlerotatus*) *vigilax* (Skuse, 1889)
**Subgenus *Paraedes* Edwards, 1934 (2 spp.)**
*Aedes* (*Paraedes*) *ostentatio* (Leicester, 1908) *Aedes* (*Paraedes*) *thailandensis* Reinert, 1976
**Subgenus *Petermattinglyius* Reinert, Harbach & Kitching, 2009 (2 spp.)**
*Aedes* (*Petermattinglyius*) *franciscoi* Mattingly, 1959 **NEW RECORD** *Aedes* (*Petermattinglyius*) *scanloni* Reinert, 1970
**Subgenus *Phagomyia* Theobald, 1905 (5 spp.)**
*Aedes* (*Phagomyia*) *assamensis* (Theobald, 1908) *Aedes* (*Phagomyia*) *feegradei* (Barraud, 1934) *Aedes* (*Phagomyia*) *khazani* Edwards, 1922 *Aedes* (*Phagomyia*) *lophoventralis* (Theobald, 1910) **NEW RECORD** *Aedes* (*Phagomyia*) *prominens* (Barraud, 1923)
**Subgenus *Scutomyia* Theobald, 1904 (1 sp.)**
*Aedes* (*Scutomyia*) *albolineatus* (Theobald, 1904)
**Subgenus *Stegomyia* Theobald, 1901 (13 spp.)**
*Aedes* (*Stegomyia*) *aegypti* (Linnaeus, 1762) *Aedes* (*Stegomyia*) *albopictus* (Skuse, 1895) *Aedes* (*Stegomyia*) *annandalei* (Theobald, 1910) *Aedes* (*Stegomyia*) *craggi* (Barraud, 1923) **NEW RECORD** *Aedes* (*Stegomyia*) *desmotes* (Giles, 1904) *Aedes* (*Stegomyia*) *imitator* (Leicester, 1908) *Aedes* (*Stegomyia*) *malayensis* Colless, 1962 *Aedes* (*Stegomyia*) *malikuli* Huang, 1973 *Aedes* (*Stegomyia*) *patriciae* Mattingly, 1954 **NEW RECORD** *Aedes (Stegomyia) pseudalbopictus* (Borel, 1928) *Aedes* (*Stegomyia*) *scutellaris* (Walker, 1859) *Aedes* (*Stegomyia*) *unalom* Maquart & Hide, 2024 *Aedes* (*Stegomyia*) *w-albus* (Theobald, 1905)
**Subgenus *Tanakaius* Reinert, Harbach & Kitching, 2004 (1 sp.)**
*Aedes* (*Tanakaius*) *togoi* (Theobald, 1907)
**Subgenus *Tewarius* Reinert, 2006 (1 sp.) NEW RECORD**
*Aedes* (*Tewarius*) *pseudonummatus* Reinert, 1973 **NEW RECORD**
**Subgenus *Rhinoskusea* Edwards, 1929 (1 sp.)**
*Aedes* (*Rhinoskusea*) *longirostris* (Leicester, 1908)
**Genus *Armigeres* Theobald, 1901 (26 spp.)**
**Subgenus *Armigeres* Theobald, 1901 (12 spp.)**
*Armigeres* (*Armigeres*) *aureolineatus* (Leicester, 1908) *Armigeres* (*Armigeres*) *confusus* Edwards, 1915 *Armigeres* (*Armigeres*) *durhami* (Edwards, 1917) *Armigeres* (*Armigeres*) *foliatus* Brug, 1931 *Armigeres* (*Armigeres*) *kesseli* Ramalingam, 1987 *Armigeres* (*Armigeres*) *kuchingensis* Edwards, 191 *Armigeres* (*Armigeres*) *laoensis* Toma & Miyagi, 2003 *Armigeres* (*Armigeres*) *malayi* (Theobald, 1901) *Armigeres* (*Armigeres*) *maximus* Edwards, 1922 *Armigeres* (*Armigeres*) *moultoni* Edwards, 1914 *Armigeres* (*Armigeres*) *subalbatus* (Coquillett, 1898) *Armigeres* (*Armigeres*) *theobaldi* Barraud, 1934
**Subgenus *Leicesteria* Theobald, 1904 (14 spp.)**
*Armigeres* (*Leicesteria*) *annulipalpis* (Theobald, 1910) *Armigeres* (*Leicesteria*) *annulitarsis* (Leicester, 1908) *Armigeres* (*Leicesteria*) *balteatus* Macdonald, 1960 *Armigeres* (*Leicesteria*) *cingulatus* (Leicester, 1908) *Armigeres* (*Leicesteria*) *dentatus* Barraud, 1927 *Armigeres* (*Leicesteria*) *digitatus* (Edwards, 1914) *Armigeres* (*Leicesteria*) *dolichocephalus* (Leicester, 1908) *Armigeres* (*Leicesteria*) *flavus* (Leicester, 1908) *Armigeres* (*Leicesteria*) *inchoatus* Barraud, 1927 *Armigeres* (*Leicesteria*) *longipalpis* (Leicester, 1904) *Armigeres* (*Leicesteria*) *magnus* (Theobald, 1908) *Armigeres* (*Leicesteria*) *omissus* (Edwards, 1914) *Armigeres* (*Leicesteria*) *pectinatus* (Edwards, 1914) *Armigeres* (*Leicesteria*) *traubi* Macdonald, 1960
**Genus *Heizmannia* Ludlow, 1905 (11 spp.)**
**Subgenus *Heizmannia* Ludlow, 1905 (9 spp.)**
*Heizmannia* (*Heizmannia*) *aureochaeta* (Leicester, 1908) *Heizmannia* (*Heizmannia*) *chengi* Lien, 1968 *Heizmannia* (*Heizmannia*) *communis* (Leicester, 1908) *Heizmannia* (*Heizmannia*) *complex* (Theobald, 1910) *Heizmannia* (*Heizmannia*) *covelli* Barraud, 1929 **NEW RECORD** *Heizmannia* (*Heizmannia*) *demeilloni* Mattingly, 1970 *Heizmannia* (*Heizmannia*) *mattinglyi* Thurman, 1959 *Heizmannia* (*Heizmannia*) *reidi* Mattingly, 1957 *Heizmannia* (*Heizmannia*) *scintillans* Ludlow, 1905
**Subgenus *Mattinglyia* Lien, 1968 (2 spp.)**
*Heizmannia* (*Mattinglyia*) *achaetae* (Leicester, 1908) *Heizmannia* (*Mattinglyia*) *catesi* Lien, 1968
**Genus *Verrallina* Theobald, 1903 (19 spp.)**
**Subgenus *Harbachius* Reinert, 1999 (5 spp.)**
*Verrallina* (*Harbachius*) *abdita* (Barraud, 1931) *Verrallina* (*Harbachius*) *fragilis* Leicester, 1908 *Verrallina* (*Harbachius*) *indecorabilis* Leicester, 1908 *Verrallina* (*Harbachius*) *stunga* (Klein, 1973) *Verrallina* (*Harbachius*) *yusafi* (Barraud, 1931)
**Subgenus *Neomacleaya* Theobald, 1907 (12 spp.)**
*Verrallina* (*Neomacleaya*) *adusta* (Laffoon, 1946) *Verrallina* (*Neomacleaya*) *andamanensis* (Edwards, 1922) *Verrallina* (*Neomacleaya*) *clavata* (Barraud, 1931) *Verrallina* (*Neomacleaya*) *cretata* (Delfinado, 1967) *Verrallina* (*Neomacleaya*) *cyrtolabis* (Edwards, 1928) *Verrallina* (*Neomacleaya*) *komponga* (Klein, 1973) *Verrallina* (*Neomacleaya*) *notabilis* (Delfinado, 1967) *Verrallina* (*Neomacleaya*) *phnoma* (Klein, 1973) *Verrallina* (*Neomacleaya*) *rara* (Delfinado, 1968) *Verrallina* (*Neomacleaya*) *taeniata* (Leicester, 1908) *Verrallina* (*Neomacleaya*) *unca* (Theobald, 1901) *Verrallina* (*Neomacleaya*) *vallistris* (Barraud, 1928)
**Subgenus *Verrallina* Theobald, 1903 (3 spp.)**
*Verrallina* (*Verrallina*) *butleri* (Theobald, 1901) *Verrallina* (*Verrallina*) *dux* (Dyar & Shannon, 1925) *Verrallina* (*Verrallina*) *lugubris* (Barraud, 1928) **NEW RECORD**
**Genus *Culex* Linnaeus, 1758 (56 spp.)**
**Subgenus *Culex* Linnaeus, 1758 (15 spp.)**
*Culex* (*Culex*) *alienus* Colless, 1957 *Culex* (*Culex*) *alis* Theobald, 1903 *Culex* (*Culex*) *barraudi* Edwards, 1922 *Culex* (*Culex*) *fuscocephala* Theobald, 1907 *Culex* (*Culex*) *gelidus* Theobald, 1901 *Culex* (*Culex*) *hutchinsoni* Barraud, 1924 *Culex* (*Culex*) *mimulus* Edwards, 1915 (dubious) *Culex* (*Culex*) *perplexus* Leicester, 1908 *Culex* (*Culex*) *pseudovishnui* Colless, 1957 *Culex* (*Culex*) *quinquefasciatus* Say, 1823 *Culex* (*Culex*) *sitiens* Wiedemann, 1828 *Culex* (*Culex*) *tritaeniorhynchus* Giles, 1901 *Culex* (*Culex*) *vishnui* Theobald, 1901 *Culex* (*Culex*) *whitei* Barraud, 1923 *Culex* (*Culex*) *whitmorei* (Giles, 1904)
**Subgenus *Culiciomyia* Theobald, 1907 (10 spp.)**
*Culex* (*Culiciomyia*) *bailyi* Barraud, 1934 *Culex (Culiciomyia) dispectus* Bram, 1966 **NEW RECORD** *Culex* (*Culiciomyia*) *fragilis* Ludlow, 1903 *Culex* (*Culiciomyia*) *nigropunctatus* Edwards, 1926 *Culex* (*Culiciomyia*) *pallidothorax* Theobald, 1905 *Culex* (*Culiciomyia*) *papuensis* (Taylor, 1914) *Culex* (*Culiciomyia*) *scanloni* Bram, 1967 *Culex* (*Culiciomyia*) *spathifurca* (Edwards, 1915) *Culex* (*Culiciomyia*) *termi* Thurman, 1955 *Culex* (*Culiciomyia*) *thurmanorum* Bram, 1967
**Subgenus *Eumelanomyia* Theobald, 1909 (11 spp.)**
*Culex* (*Eumelanomyia*) *bokorensis* Klein & Sirivanakarn, 1970 *Culex* (*Eumelanomyia*) *brevipalpis* (Giles, 1902) *Culex* (*Eumelanomyia*) *foliatus* Brug, 1932 *Culex* (*Eumelanomyia*) *hinglungensis* Chu, 1957 *Culex* (*Eumelanomyia*) *kiriensis* Klein & Sirivanakarn, 1970 *Culex* (*Eumelanomyia*) *malayi* (Leicester, 1908) *Culex* (*Eumelanomyia*) *oresbius* Harbach & Rattanarithikul, 1988 *Culex* (*Eumelanomyia*) *otachati* Klein & Sirivanakarn, 1970 *Culex* (*Eumelanomyia*) *richei* Klein, 1970 *Culex* (*Eumelanomyia*) *selai* Klein & Sirivanakarn, 1970 *Culex* (*Eumelanomyia*) *tenuipalpis* Barraud, 1924
**Subgenus *Lophoceraomyia* Theobald, 1905 (18 spp.)**
*Culex* (*Lophoceraomyia*) *aculeatus* Colless, 1965 *Culex* (*Lophoceraomyia*) *cinctellus* Edwards, 1922 *Culex* (*Lophoceraomyia*) *curtipalpis* (Edwards, 1914) *Culex* (*Lophoceraomyia*) *eukrines* Bram & Rattanarithikul, 1967 *Culex* (*Lophoceraomyia*) *fraudatrix* (Theobald, 1905) *Culex* (*Lophoceraomyia*) *ganapathi* Colless, 1965 *Culex* (*Lophoceraomyia*) *inculus* Colless, 1965 *Culex* (*Lophoceraomyia*) *infantulus* Edwards, 1922 *Culex* (*Lophoceraomyia*) *macdonaldi* Colless, 1965 *Culex* (*Lophoceraomyia*) *mammilifer* (Leicester, 1908) *Culex* (*Lophoceraomyia*) *minor* (Leicester, 1908) *Culex* (*Lophoceraomyia*) *peytoni* Bram & Rattanarithikul, 1967 *Culex* (*Lophoceraomyia*) *quadripalpis* (Edwards, 1914) *Culex* (*Lophoceraomyia*) *reidi* Colless, 1965 *Culex* (*Lophoceraomyia*) *rubithoracis* (Leicester, 1908) *Culex* (*Lophoceraomyia*) *spiculosus* Bram & Rattanarithikul, 1967 *Culex* (*Lophoceraomyia*) *variatus* (Leicester, 1908) *Culex* (*Lophoceraomyia*) *wilfredi* Colless, 1965
**Subgenus *Oculeomyia* Theobald, 1907 (4 spp.)**
*Culex* (*Oculeomyia*) *bitaeniorhynchus* Giles, 1901 *Culex* (*Oculeomyia*) *infula* Theobald, 1901 **NEW RECORD** *Culex* (*Oculeomyia*) *pseudosinensis* Colless, 1955 *Culex* (*Oculeomyia*) *sinensis* Theobald, 1903
**Genus *Culiseta* Felt, 1904 (1 sp.)**
**Subgenus *Climacura* Howard, Dyar & Knab, 1915 (1 sp.)**
*Culiseta* (*Climacura*) *marchettei* Garcia, Jeffery & Rudnick, 1969 (dubious)
**Genus *Lutzia* Theobald, 1903 (3 spp.)**
**Subgenus *Metalutzia* Tanaka, 2003 (3 spp.)**
*Lutzia* (*Metalutzia*) *fuscana* (Wiedemann, 1820) *Lutzia* (*Metalutzia*) *halifaxii* (Theobald, 1903) *Lutzia* (*Metalutzia*) *vorax* Edwards, 1921
**Genus *Ficalbia* Theobald, 1903 (1 sp.)**
*Ficalbia minima* (Theobald, 1901)
**Genus *Mimomyia* Theobald, 1903 (7 spp.)**
**Subgenus *Etorleptiomyia* Theobald, 1904 (2 spp.)**
*Mimomyia* (*Etorleptiomyia*) *elegans* (Taylor, 1914) *Mimomyia* (*Etorleptiomyia*) *luzonensis* (Ludlow, 1905)
**Subgenus *Ingramia* Edwards, 1912 (2 spp.)**
*Mimomyia* (*Ingramia*) *fusca* (Leicester, 1908) *Mimomyia* (*Ingramia*) *kiriromi* (Klein, 1969)
**Subgenus *Mimomyia* Theobald, 1904 (3 spp.)**
*Mimomyia* (*Mimomyia*) *aurea* (Leicester, 1908) *Mimomyia* (*Mimomyia*) *chamberlaini* Ludlow, 1904 *Mimomyia* (*Mimomyia*) *hybrida* (Leicester, 1908)
**Genus *Hodgesia* Theobald, 1904 (3 spp.)**
**Subgenus *Hodgesia* Theobald, 1904 (3 spp.)**
*Hodgesia* (*Hodgesia*) *bailyi* Barraud, 1929 *Hodgesia* (*Hodgesia*) *malayi* Leicester, 1908 *Hodgesia* (*Hodgesia*) *quasisanguinae* Leicester, 1908
**Genus *Coquillettidia* Dyar, 1905 (3 spp.)**
**Subgenus *Coquillettidia* Dyar, 1905 (3 spp.)**
*Coquillettidia* (*Coquillettidia*) *crassipes* (van der Wulp, 1881) *Coquillettidia* (*Coquillettidia*) *nigrosignata* (Edwards, 1917) *Coquillettidia* (*Coquillettidia*) *ochracea* (Theobald, 1903)
**Genus *Mansonia* Blanchard, 1901 (6 spp.)**
**Subgenus *Mansonioides* Theobald, 1907 (6 spp.)**
*Mansonia* (*Mansonioides*) *annulata* Leicester, 1908 **NEW RECORD** *Mansonia* (*Mansonioides*) *annulifera* (Theobald, 1901) *Mansonia* (*Mansonioides*) *bonneae* Edwards, 1930 *Mansonia* (*Mansonioides*) *dives* (Schiner, 1868) *Mansonia* (*Mansonioides*) *indiana* Edwards, 1930 *Mansonia* (*Mansonioides*) *uniformis* (Theobald, 1901)
**Genus *Orthopodomyia* Theobald, 1904 (4 spp.)**
*Orthopodomyia* (*Orthopodomyia*) *andamanensis* Barraud, 1934 *Orthopodomyia* (*Orthopodomyia*) *anopheloides* (Giles, 1903) *Orthopodomyia* (*Orthopodomyia*) *siamensis* Zavortink, 1968 *Orthopodomyia* (*Orthopodomyia*) *wilsoni* Macdonald, 1958 **NEW RECORD**
**Genus *Malaya* Leicester, 1908 (2 spp.)**
*Malaya* (*Malaya*) *genurostris* Leicester, 1908 *Malaya* (*Malaya*) *jacobsoni* (Edwards, 1930)
**Genus *Topomyia* Leicester, 1908 (4 spp.)**
**Subgenus *Topomyia* Leicester, 1908 (4 spp.)**
*Topomyia* (*Topomyia*) *angkoris* Klein, 1977 *Topomyia* (*Topomyia*) *apsarae* Klein, 1977 *Topomyia* (*Topomyia*) *argyropalpis* Leicester, 1908 *Topomyia* (*Topomyia*) *gracilis* Leicester, 1908
**Genus *Tripteroides* Giles, 1904 (6 spp.)**
**Subgenus *Rachionotomyia* Theobald, 1905 (2 spp.)**
*Tripteroides* (*Rachionotomyia*) *affinis* (Edwards, 1913) *Tripteroides* (*Rachionotomyia*) *aranoides* (Theobald, 1901)
**Subgenus *Tripteroides* Giles, 1904 (4 spp.)**
*Tripteroides* (*Tripteroides*) *aeneus* (Edwards, 1921) *Tripteroides* (*Tripteroides*) *caeruleocephalus* (Leicester, 1908) *Tripteroides* (*Tripteroides*) *powelli* (Ludlow, 1909) *Tripteroides* (*Tripteroides*) *similis* (Leicester, 1908)
**Genus *Toxorhynchites* Theobald, 1901 (5 spp.)**
**Subgenus *Toxorhynchites* Theobald, 1901 (5 spp.)**
*Toxorhynchites* (*Toxorhynchites*) *albipes* (Edwards, 1922) *Toxorhynchites* (*Toxorhynchites*) *domrey* Maquart & Rahola, 2023 *Toxorhynchites* (*Toxorhynchites*) *gravelyi* (Edwards, 1921) *Toxorhynchites* (*Toxorhynchites*) *kempi* (Edwards, 1921) *Toxorhynchites* (*Toxorhynchites*) *splendens* (Wiedemann, 1819)
**Genus *Uranotaenia* Lynch Arribálzaga, 1891 (28spp.)**
**Subgenus *Pseudoficalbia* Theobald, 191*2* (14 spp.)**
*Uranotaenia* (*Pseudoficalbia*) *abdita* Peyton, 1977 *Uranotaenia* (*Pseudoficalbia*) *albipes* Peyton, 1977 *Uranotaenia* (*Pseudoficalbia*) *bicolor* Leicester, 1908 *Uranotaenia* (*Pseudoficalbia*) *demeilloni* Peyton & Rattanarithikul, 1970 **NEW RECORD** *Uranotaenia* (*Pseudoficalbia*) *gouldi* Peyton & Klein, 1970 *Uranotaenia* (*Pseudoficalbia*) *hirsutifemora* Peters, 1964 *Uranotaenia* (*Pseudoficalbia*) *koli* Peyton & Klein, 1970 *Uranotaenia* (*Pseudoficalbia*) *lutescens* Leicester, 1908 *Uranotaenia* (*Pseudoficalbia*) *maxima* Leicester, 1908 *Uranotaenia* (*Pseudoficalbia*) *nivipleura* Leicester, 1908 *Uranotaenia* (*Pseudoficalbia*) *novobscura* Barraud, 1934 *Uranotaenia* (*Pseudoficalbia*) *obscura* Edwards, 1915 *Uranotaenia* (*Pseudoficalbia*) *pseudomaculipleura* Peyton & Rattanarithikul, 1970 *Uranotaenia* (*Pseudoficalbia*) *spiculosa* Peyton & Rattanarithikul, 1970
**Subgenus *Uranotaenia* Lynch Arribálzaga, 1891 (14 spp.)**
*Uranotaenia* (*Uranotaenia*) *annandalei* Barraud, 1926 *Uranotaenia* (*Uranotaenia*) *bimaculiala* Leicester, 1908 *Uranotaenia* (*Uranotaenia*) *campestris* Leicester, 1908 *Uranotaenia* (*Uranotaenia*) *diraphati* Peyton & Klein, 1970 *Uranotaenia* (*Uranotaenia*) *lateralis* Ludlow, 1905 *Uranotaenia* (*Uranotaenia*) *longirostris* Leicester, 1908 *Uranotaenia* (*Uranotaenia*) *macfarlanei* Edwards, 1914 *Uranotaenia* (*Uranotaenia*) *metatarsata* Edwards, 1914 *Uranotaenia* (*Uranotaenia*) *micans* Leicester, 1908 *Uranotaenia* (*Uranotaenia*) *rampae* Peyton & Klein, 1970 *Uranotaenia* (*Uranotaenia*) *sombooni* Peyton & Klein, 1970 *Uranotaenia* (*Uranotaenia*) *subnormalis* Martini, 1920 *Uranotaenia* (*Uranotaenia*) *testacea* Theobald, 1905 *Uranotaenia* (*Uranotaenia*) *trilineata* Leicester, 1908

### Genus *Aedes* Meigen, 1818

#### Subgenus *Aedimorphus* Theobald, 1903

##### *Aedes (Aedimorphus) nigrostriatus* (Barraud, 1927)

Material examined: 3**♀♀** Cambodia, Mondulkiri province, Kaoh Nheaek district, Roveak, 13.159992 N, 106.898913 E, 10.VIII.2020, 147 m, collected at 08 PM using a double net trap, with a human bait; Institut Pasteur du Cambodge, Malaria Research Unit. 8**♀♀** same data but collection time: 09 PM.

Species previously known from India and Myanmar [15, 25].

#### Subgenus *Bruceharrisonius* Reinert, 2003

##### *Aedes* (*Bruceharrisonius*) *greenii* (Theobald, 1903)

Material examined: 1**♀** Cambodia, Mondulkiri province, Kaev Seima district, 12.28507472 N; 106.99360712 E; X.2021; 338 m, collected at 09 AM using a double net trap, with a human bait; Institut Pasteur du Cambodge, Malaria Research Unit.

Species previously known from India, Indonesia, Laos, Malaysia, Nepal, Philippines, Sri Lanka, and Thailand [10, 15, 25].

#### Subgenus *Downsiomyia* Vargas, 1950

##### *Aedes* (*Downsiomyia*) *ganapathi* Colless, 1958

Material examined: 1**♀** Cambodia, Mondulkiri province, Kaoh Nheaek district, Sector 31, 13.136553 N, 106.858282 E, 17.VII.2020, 169 m, collected at 06 PM using a double net trap, with a human bait; Institut Pasteur du Cambodge, Malaria Research Unit.

Species previously known from Laos, Malaysia, and Thailand [10, 15, 25].

##### *Aedes* (*Downsiomyia*) *harinasutai* Knight, 1978

Material examined: 2**♀♀** Cambodia, Mondulkiri province, Kaoh Nheaek district, Sector 31, 13.136553 N, 106.858282 E, 16.VI.2020, 169 m, collected at 02 PM using a double net trap, with a human bait; Institut Pasteur du Cambodge, Malaria Research Unit.

Species previously known from Laos and Thailand [10, 15, 25].

##### *Aedes* (*Downsiomyia*) *nipponicus* La Casse & Yamaguti, 1948

Material examined: 1**♀** Cambodia, Mondulkiri province, Kaoh Nheaek district, Sector 07, 13.140144 N, 106.790236 E, 16.VII.2020, 178 m, collected at 10 PM using a double net trap, with a human bait; Institut Pasteur du Cambodge, Malaria Research Unit. 1**♀**Cambodia, Mondulkiri province, Kaoh Nheaek district, Sector 31, 13.136553 N, 106.858282 E, 15.VII.2020, 169 m, collected at 07 PM using a double net trap, with a human bait; Institut Pasteur du Cambodge, Malaria Research Unit.

Species previously known from Japan, Russia, South Korea, and Thailand [15, 25].

##### *Aedes* (*Downsiomyia*) *novoniveus* Barraud, 1934

Material examined: 3**♀♀** Cambodia, Mondulkiri province, Kaev Seima district, 12.28507472 N; 106.99360712 E; 15.X.2021; 338 m, collected at 09 AM using a double net trap, with a human bait; Institut Pasteur du Cambodge; Malaria Research Unit. 2 **♀** same data but collection date and time: 15.VII.2020; 03 PM.

Species previously known from India, Malaysia, Nepal, and Thailand [15, 25].

##### *Aedes* (*Downsiomyia*) *litoreus* Colless, 1958

Material examined: 1**♀** Cambodia, Mondulkiri province, Kaoh Nheaek district, Sector 07, 13.140144 N, 106.790236 E, 12.XII.2020, 178 m, collected at 04 AM using a double net trap, with a human bait; Institut Pasteur du Cambodge; Malaria Research Unit.

Species previously known from Malaysia, Singapore, and Thailand [15, 25].

#### Subgenus *Petermattinglyius* Reinert, Harbach & Kitching, 2009

##### *Aedes* (*Petermattinglyius*) *franciscoi* Mattingly, 1959

Material examined: 1**♀** Cambodia, Mondulkiri province, Kaoh Nheaek district, Sector 07, 13.140144 N, 106.790236 E, 12.XII.2020, 178 m, collected at 04 AM using a double net trap, with a human bait; Institut Pasteur du Cambodge; Malaria Research Unit.

Species previously known from Malaysia, Singapore, and Thailand [15, 25].

#### Subgenus *Phagomyia* Theobald, 1905

##### *Aedes* (*Phagomyia*) *lophoventralis* (Theobald, 1910)

Material examined: 1**♀** Cambodia, Mondulkiri province, Kaoh Nheaek district, Sector 07, 13.140144 N 106.790236 E, 16.VII.2020, 178 m, collected at 09 AM using a double net trap, with a human bait; Institut Pasteur du Cambodge; Malaria Research Unit. 1**♀**Cambodia, Mondulkiri province, Kaoh Nheaek district, Sector 22, 13.051882N, 106.674865E, 16.VII.2020, 176 m, collected at 01 PM using a double net trap, with a human bait; Institut Pasteur du Cambodge; Malaria Research Unit.

Species previously known from Bangladesh, India, Nepal, Thailand, and Vietnam [15, 25].

#### Subgenus *Stegomyia* Theobald, 1901

##### *Aedes* (*Stegomyia*) *craggi* (Barraud, 1923)

Material examined: 1**♀** Cambodia, Mondulkiri province, Kaoh Nheaek district, Sector 07, 13.140144 N, 106.790236 E, 13.XI.2020, 178 m, collected at 04 PM using a double net trap, with a human bait; Institut Pasteur du Cambodge; Malaria Research Unit.

Species previously known from Laos, India, Nepal, and Thailand [10, 15, 25].

##### *Aedes* (*Stegomyia*) *patriciae* Mattingly, 1954

Material examined: 2**♂♂** Cambodia, Mondulkiri province, Kaoh Nheaek district, Sector 07, 13.140144 N, 106.790236 E, 12.V.2020, 178 m, collected at 06 AM using a double net trap, with a human bait; Institut Pasteur du Cambodge; Malaria Research Unit. 1**♀** same data but collection date and time: 13.XI.2020; 04 PM. 1♂ Cambodia, Mondulkiri province, Kaev Seima district, 12.181324 N, 106.87287 E, 01.XI.2019, 338 m, collected using a double net trap, with a human bait.

Species previously known from India, Malaysia, Pakistan, People’s Republic of China, Thailand, and Vietnam [25].

#### Subgenus *Tewarius* Reinert, 2006

##### *Aedes* (*Tewarius*) *pseudonummatus* Reinert, 1973

Material examined: 1**♀** Cambodia, Mondulkiri province, Kaoh Nheaek district, Sector 22, 13.051882 N, 106.674865 E, 15.I.2020, 176 m, collected at 04 AM using a double net trap, with a human bait; Institut Pasteur du Cambodge; Malaria Research Unit. 1**♀**Cambodia, Mondulkiri province, Kaoh Nheaek district, Sector 36, 13.025147 N, 106.829365 E, 09.X.2020, 180 m, collected at 03 PM using a double net trap, with a human bait; Institut Pasteur du Cambodge; Malaria Research Unit.

Species previously known from Laos and Thailand [10, 15, 25].

#### Genus *Kenknightia* Reinert, 1990

##### *Aedes* (*Kenknightia*) *harbachi* Reinert, 1990

Material examined: 1**♀** Cambodia, Mondulkiri province, Kaoh Nheaek district, Sector 34, 13.025147 N, 106.829365 E, 17.VII.2020, 181 m, collected at 05 PM using a double net trap, with a human bait; Institut Pasteur du Cambodge; Malaria Research Unit. 1**♀**Cambodia, Mondulkiri province, Kaoh Nheaek district, Roveak, 13.164098 N, 106.902797 E, 21.V.2020, 147 m, collected at 02 AM using a double net trap, with a human bait; Institut Pasteur du Cambodge; Malaria Research Unit.

Species previously known from Laos, India, China, and Thailand [10, 15, 25].

### Genus *Culex* Linnaeus, 1758

#### Subgenus *Culiciomyia* Theobald, 1907

##### *Culex (Culiciomyia) dispectus* Bram, 1966

Material examined: 1**♀** Cambodia, Mondulkiri province, Kaoh Nheaek district, Sector 34, 13.025147 N, 106.829365 E, 181 m, 17.VII.2020, collected at 08 PM using a double net trap, with a human bait; Institut Pasteur du Cambodge; Malaria Research Unit.

Species previously known from Laos, Malaysia, People’s Republic of China, and Thailand [10, 17, 25].

#### Subgenus *Oculeomyia* Theobald, 1907

##### *Culex* (*Oculeomyia*) *infula* Theobald, 1901

Material examined: 1**♀** Cambodia, Mondulkiri province, Kaoh Nheaek district, Sector 34, 13.025147 N, 106.829365 E, 181 m, 17.VII.2020, collected at 08 PM using a double net trap, with a human bait; Institut Pasteur du Cambodge; Malaria Research Unit.

Species previously known from Bangladesh, India, Indonesia, Malaysia, Myanmar, Nepal, Philippines, Singapore, Sri Lanka, Thailand, and Vietnam [17, 25].

### Genus *Heizmannia* Ludlow, 1905

#### Subgenus *Heizmannia* Ludlow, 1905

##### *Heizmannia* (*Heizmannia*) *covelli* Barraud, 1929

Material examined: 1**♀** Cambodia, Mondulkiri province, Kaoh Nheaek district, Sector 31, 13.136553 N, 106.858282 E, 14.VI.2020, 169 m, collected at 07 PM using a double net trap, with a human bait; Institut Pasteur du Cambodge; Malaria Research Unit. 1**♀** same data but collection time: 11 PM. 1**♀** same data but date and collection time: 16.VI.2020; 02 PM. 1**♀**Cambodia, Mondulkiri province, Kaoh Nheaek district, Sector 34, 13.025147 N, 106.829365 E, 17.VI.2020, 181 m, collected at 01 AM using a double net trap, with a human bait; Institut Pasteur du Cambodge; Malaria Research Unit.

Species previously known from Bangladesh, India, Myanmar, Thailand, and Vietnam [17, 25].

### Genus *Verrallina* Theobald, 1903

#### Subgenus *Verrallina* Theobald, 1903

##### *Verrallina* (*Verrallina*) *lugubris* (Barraud, 1928)

Material examined: 1**♀**Cambodia, Mondulkiri province, Kaoh Nheaek district, Sector 22, 13.051882 N, 106.674865 E, 13.I.2020, 176 m, collected at 05 AM using a double net trap, with a human bait; Institut Pasteur du Cambodge; Malaria Research Unit.

Species previously known from Laos, India, Malaysia, Myanmar, and Thailand [10, 17, 25].

### Genus *Mansonia* Blanchard, 1901

#### Subgenus *Mansonioides* Theobald, 1907

##### *Mansonia* (*Mansonioides*) *annulata* Leicester, 1908

Material examined: 1**♀** Cambodia, Mondulkiri province, Kaoh Nheaek district, Roveak village, 13.160042 N, 106.898877 E, 17.VI.2020, 147 m, collected at 12 AM using a double net trap, with a human bait; Institut Pasteur du Cambodge; Malaria Research Unit. 1**♀** same data but collection date and time: 13.XI.2020; 02 PM; 1**♀**, same data but collection date: 12.XI.2020

Species previously known from Bangladesh, Indonesia, Malaysia, Philippines, Thailand, and Vietnam [17, 25].

### Genus *Orthopodomyia* Theobald, 1904

#### Subgenus *Orthopodomyia* Theobald, 1904

##### *Orthopodomyia* (*Orthopodomyia*) *wilsoni* Macdonald, 1958

Material examined: 1**♀** Cambodia, Mondulkiri province, Kaoh Nheaek district, Sector 15, 13.138208 N, 106.675692 E, 09.X.2020, collected at 10 PM using a double net trap, with a human bait; Institut Pasteur du Cambodge; Malaria Research Unit. 1**♀** Cambodia, Mondulkiri province, Kaoh Nheaek district, Sector 22, 13.051882 N, 106.674865 E, 10.X.2020, 176 m, collected at 01 AM using a double net trap, with a human bait; Institut Pasteur du Cambodge; Malaria Research Unit.

Species previously known from Malaysia and Thailand [16, 25].

### Genus *Uranotaenia* Lynch Arribálzaga, 1891

#### Subgenus *Pseudoficalbia* Theobald, 1912

##### *Uranotaenia* (*Pseudoficalbia*) *demeilloni* Peyton & Rattanarithikul, 1970

Material examined: 1**♀** Cambodia, Mondulkiri province, Kaoh Nheaek district, Sector 15, 13.138208 N, 106.675692 E, 09.X.2020, collected at 10 PM using a double net trap, with a human bait; Institut Pasteur du Cambodge; Malaria Research Unit. 1**♀** Cambodia, Mondulkiri province, Kaoh Nheaek district, Sector 22, 13.051882 N, 106.674865 E, 10.X.2020, 176 m, collected at 01 AM using a double net trap, with a human bait; Institut Pasteur du Cambodge; Malaria Research Unit.

Species previously known from Laos, Malaysia, Philippines, Thailand, and Vietnam [10, 16, 25].

## Discussion

In total, thirteen of the newly recorded species belong to the *Aedes* genus. This can be explained by the difficulty in identifying some of the species. Most of them were probably already collected in our previous studies, but could not be properly identified given their conservation state. The double net collection technique makes it possible to collect better quality mosquitoes, facilitating their identification. Additionally, our collection methods (either double-net trap in the present article, or CDC-light trap and BG traps in previous ones) allowed us to collect mostly female specimens, sometimes making identification more challenging than on males. While we previously described 43 species considered to be of medical or veterinary importance [8], no new medically important vectors are reported in the present update [25]. However, it is important to highlight that the collection method used in the present study (double net trapping) allows for the collection of mosquitoes attracted to human odor, meaning that they would probably feed on humans. This is important to considered as a potential route of emergence of new pathogens. Most of the feeding habits of these species are yet unknown [8].

Overall, since 2016, the Medical and Veterinary Entomology Unit of the *Institut Pasteur du Cambodge* has collected over 600,000 mosquitoes belonging to 312 species from 16 genera. Previously, this unprecedented sampling effort allowed us to increase previous estimates from 241 species to 290 species [8]. Recently, two new species have been described from Cambodia [3, 9], highlighting the lack of knowledge in the country. As mentioned in previous work [8], the province of Mondulkiri was investigated more in-depth, explaining this increase in number, but further collection efforts should concentrate on areas like the Cardamom, Kampot, or the Aoral mountains, as well as the forests in Rattanakiri and Mondulkiri, which host some of the most biologically diverse biomes in the country [9]. Given that Cambodia has one of the world’s highest deforestation rates [1, 11, 23], the situation is alarming: interfaces between anthropic areas and forests are rapidly blurring, increasing the risk of the population being exposed to new emergent diseases and vectors. This also underscores the need to inventory Culicidae fauna and their associated pathogens as much as possible.

## Conclusion

The information generated in this study refines our understanding of mosquito diversity in Cambodia, bringing the total number of recorded species to 312, from 16 genera, with 43 species of medical or veterinary importance. Given Cambodia’s increasing human-wildlife interface, the risk of emerging vector-borne diseases is rising. The presence of numerous species attracted to humans emphasizes the urgent need for continued surveillance. Systematic inventories and research are essential to anticipate and mitigate future public health threats.
